# Exploring consumer responses to official endorsement: roles of credibility and attractiveness attributes in live streaming

**DOI:** 10.3389/fpsyg.2024.1371343

**Published:** 2024-05-20

**Authors:** Guo Cheng, Wenjie Li, Mingli He, Luyuan Liao

**Affiliations:** ^1^Faculty of Economics and Business Administration, Yibin University, Yibin, China; ^2^School of Business, Sun Yat-sen University, Guangzhou, China; ^3^Business School, Sichuan University, Chengdu, China

**Keywords:** official credibility attributes, official attractiveness attributes, perceived security, perceived enjoyment, purchase intention, local brand awareness

## Abstract

**Introduction:**

Official endorsement, distinct from celebrity, expertise, and peer endorsement, introduces a new paradigm where local government officials use online platforms, particularly live streaming, to promote local products and brands.

**Methods:**

This study examines the influence of official endorsement on consumer responses using the source credibility and source attractiveness models. We developed a framework that considers official credibility and attractiveness attributes as antecedents, and consumer perceived security and enjoyment as mediators, affecting purchase intention and local brand awareness. The study also incorporates variables such as consumer region and power distance belief.

**Results:**

Data from 594 responses obtained through an online survey were analyzed using structural equation modeling. The results indicate that official credibility attributes (expertise, trustworthiness, government credibility) enhances consumer perceived security, while official attractiveness attributes (physical attractiveness, interaction friendliness, and similarity with consumers) increases consumer enjoyment. Both perceived security and enjoyment positively influence purchase intention and local brand awareness. These relationships are partially moderated by consumer region and power distance belief.

**Discussion:**

This research pioneers the study of official endorsements, expanding the endorsement literature. It also provides practical insights for marketing professionals and government officials on leveraging official endorsements to enhance the value of local products and brands..

## 1 Introduction

Endorsement is a pivotal marketing strategy for practitioners, encompassing explicit forms (e.g., “I endorse this product/brand”) and implicit forms (e.g., “I use this product/brand”) or simply appearing alongside a product/brand in specific settings (McCracken, 1989; Bush et al., 2004; Halder et al., 2021). Effective endorsement enhances consumer attitudes toward advertisements, products, and brands, fosters brand association or awareness, and drives purchase intention along with other positive behavioral responses (Shan et al., 2020; Zhang et al., 2021).

With the rapid advancement of internet technology and the local government’s emphasis on regional economic growth, many local government officials have begun to endorse local products and brands via online platforms, particularly through live streaming. For example, Jiaolong He, Deputy Director of the Cultural Tourism Bureau, promoted Yili’s products on the Douyin live streaming platform on behalf of Yili Kazak Autonomous Prefecture, Xinjiang, China. Similarly, Le Yang, Deputy Magistrate of Langao County, Shaanxi Province, China, endorsed Langao’s goods on Douyin and Weibo. As legitimate representatives of their regions, government officials aim to influence consumers through their authority and personal charm. Besides boosting sales of local products (Carlson et al., 2020), official endorsement aids in building regional brands and stimulating the local economy, serving as a beneficial economic strategy for local governments. Consequently, local brands and marketing practitioners must comprehend the workings of official endorsement and learn how to leverage it for successful marketing outcomes.

While extensive research has been conducted on endorsements (von and Breuer, 2020; Lili et al., 2022; Choi et al., 2023), studies focusing specifically on official endorsements are notably limited. Endorsers are essential to the endorsement process, significantly influencing outcomes alongside product-related factors and the compatibility between the endorser and the product (Halder et al., 2021). This holds particularly true for official endorsements, where local government officials, serving as authentic representatives of local products and brands, can profoundly impact public consumer perceptions through interpersonal charisma (John et al., 2019). In response, this study delves into the personal attributes of officials—such as physical attractiveness, trustworthiness, and interaction friendliness—to discern their effects on consumer responses.

Endorsements are not without risks, as illustrated by incidents where social media influencers have endorsed products without adequate vetting, later revealing significant quality issues (Deshbhag and Mohan, 2020). Especially following public safety crises, there is an increased focus on safety, including both physical and psychological aspects (Wisutwattanasak et al., 2023). Official endorsements, regulated and scrutinized by government entities, typically offer a higher security guarantee, potentially enhancing consumers’ perceptions of security. Moreover, when officials, traditionally perceived as distant or serious, engage directly with the public on the internet to endorse local products, they disrupt their conventional image, fostering a closer connection with the community and enhancing positive public evaluations. Building on the source credibility theory (Hovland et al., 1953) and source attractiveness theory (McGuire, 1985), this study aims to assess the effects of official credibility and attractiveness attributes on consumers’ perceived security and enjoyment. It further explores how these perceptions influence purchase intention and local brand awareness. Additionally, this research considers unique consumer characteristics, such as regional background (local consumers vs. out-shoppers) and power distance belief, to provide a comprehensive understanding of official endorsements.

This study makes several notable contributions to the field. Firstly, it delves into the reasons behind consumer responses to official endorsements, providing a theoretical framework that extends our understanding of how official endorsements influence consumer behavior (Janssen et al., 2022; Tian and Li, 2022; Choi et al., 2023). Secondly, this research contributes to the theoretical framework of source credibility theory and source attractiveness theory (Hovland et al., 1953; McGuire, 1985; Schimmelpfennig and Hunt, 2020; Halder et al., 2021). While expertise and trustworthiness have traditionally been considered critical sources of credibility (Hovland et al., 1953; Ohanian, 1990), this study introduces government credibility as another crucial source of official credibility. Moreover, likeability, similarity, and familiarity were regarded as main sources of attractiveness (McGuire, 1985). This study expands the concept of official attractiveness within the context of official endorsements, delineating three sources: physical attractiveness, interaction friendliness, and similarity with consumers. Then, this study delves into the differential effects of official credibility and attractiveness attributes on consumers’ security and enjoyment perceptions, as well as their effects on purchase intention and local brand awareness. By exploring these nuanced relationships, the study advances the existing literature on endorsement. Finally, this study further contributes to the literature on consumer types and government-related issues, offering new insights into how and when governmental actions influence consumer perceptions and behaviors (Kim et al., 2019; Arshad and Khurram, 2020).

## 2 Literature review

### 2.1 Endorsement and official endorsement

#### 2.1.1 Endorsement platforms and live streaming endorsement

Traditional endorsement primarily utilizes mass media like TV and radio (Ding and Qiu, 2017), where celebrities and other endorsers promote brands in a static format. In recent years, internet and mobile technologies have propelled social media, particularly live streaming, as new platforms for information, entertainment, and product purchases.

The unique appeal of live streaming lies in its video content and real-time communication (Rungruangjit, 2022). Unlike TV endorsements, live streaming offers a more vivid and dynamic approach, allowing streamers to present product features and prices and respond to consumers’ real-time inquiries and demands. This dynamic interaction enables consumers to learn detailed product information and develop parasocial relationships with streamers (Lim et al., 2020), potentially stimulating purchase and engagement behaviors (Xu et al., 2020).

Consequently, live streaming has become a popular marketing channel for selling products and promoting brands (Li et al., 2023). Live streaming enhances the role of streamers, leading to an increase in celebrities using it to endorse and sell products (Meng et al., 2021). Even local government officials have begun using live streaming to promote local brands. Numerous studies have dedicated attention to the attributes of live streamers, with a primary focus on factors such as trustworthiness, attractiveness, and expertise (Qiu et al., 2021). These studies have underscored the positive impacts of live streamers’ attributes on various aspects of consumers, including emotional attachment (Li and Peng, 2021), value perception, purchase intention (Guo et al., 2022), and other psychological states and behaviors.

#### 2.1.2 Endorser types and official endorser

Companies must strategically select endorsers, such as celebrities, experts, entrepreneurs, consumers, or politicians to fulfill their marketing objectives (McCracken, 1989). Celebrity endorsement, the most prevalent form, involves high-profile individuals like singers, actors, athletes, and politicians promoting products and brands (Schimmelpfennig and Hunt, 2020). Various studies have explored the differences among these endorsers. Biswas et al. (2006) analyzed how celebrity and expert endorsements impact consumer risk perception differently, Wang (2006) compared the impacts of expert and consumer endorsements on audience responses, and Shi et al. (2021) discussed the endorsement effectiveness between online star and traditional celebrity. Table 1 discusses the differences among various types of endorsers.

**Table 1:** Comparison of the types of endorsers.

*Type of endorsers*	*Uniqueness*	*Factors affecting consumers*	*Sources*
Celebrity (actor, singer, athlete, and politician)	High public recognition	Trustworthiness; Expertise; Attractiveness; Similarity; Familiarity; Match-up congruence with brand/product	Ha and Lam (2016)
Online celebrity	High public recognition; Similarity with public consumers	Physical attractiveness; Expertness; Social interaction; Content; Similarity with public consumers	Shi et al. (2021)
Expert	Rich knowledge and experiences in a particular domain	Expertness	Biswas et al. (2006)
Consumer/peer	Similarity with consumers	Trustworthiness; Expertness; Similarity; Attractiveness	Munnukka et al. (2016)
Official	Government credibility	Official characteristics; Government credibility; Product characteristics	He et al. (2022)
Entrepreneur	Authority in the organization; Expertise in a particular domain	Attractiveness; Expertise; Trustworthiness; Congruence	Teng et al. (2020)

This study defines official endorsement as the promotion of local products and brands by local government officials aimed at the collective benefit of the local area. Unlike other endorsers, local government officials, vested with legitimate authority, represent local communities or regions and engage in activities for collective benefit (John et al., 2019). Their endorsements are primarily nonprofit and politically oriented (Clayman, 2017), and an official’s image has been shown to influence the local area’s image (Nicolau et al., 2020). The significant role of official endorsers in local brand endorsement necessitates an exploration of the factors influencing consumer behavior in response to official endorsement. He et al. (2022) concentrated on official live streaming and proposed that characteristics of product and officer streamer would affect consumer engagement behavior and purchase intention. However, their study overlooked the impact of official endorsement on local brands. We utilize theories from the source endorsement effectiveness theory to address this gap, focusing on the significance of official endorsement for promoting local brands.

### 2.2 Source endorsement effectiveness model

#### 2.2.1 Source credibility model

Source credibility refers to an individual’s perception of an information source’s believability (O’keefe, 2015; Halder et al., 2021). Expertise and trustworthiness are the fundamental dimensions of the source credibility model (Hovland et al., 1953; Ohanian, 1990). Expertise is the extent to which a communicator is perceived as a reliable source of information, while trustworthiness is the degree of confidence in the communicator’s intent to convey valid assertions. Compared to other forms of endorsement, government involvement strongly influences official endorsement. Even when consumers find assessing an official’s competence and intentions challenging, the government’s credibility can instill a sense of security. Government credibility reflects the public’s trust in the government, encompassing citizens’ judgments about its capabilities and intentions (Zhang et al., 2019). The government’s guarantee is an undeniable unique advantage of official endorsers. Consequently, this study proposes a three-dimensional conceptualization of official credibility for official endorsement, encompassing official expertise, trustworthiness, and government credibility.

#### 2.2.2 Source attractiveness model

Source attractiveness is defined as the degree to which information receivers find the source appealing (Kiecker and Cowles, 2002; Teng et al., 2014). This attractiveness encompasses not only physical appeal but also mental skills, personality, lifestyle, and artistic talents (Erdogan, 1999). McGuire (1985) posits that source attractiveness includes similarity, familiarity, and likeability, suggesting that sources known, liked, or similar to consumers are attractive and persuasive. Similarity refers to the perceived resemblance between the source and receiver in demographic or ideological aspects, such as values and interests (Munnukka et al., 2016; Lou and Yuan, 2019). Familiarity denotes the extent of information the receiver possesses about the source (Munnukka et al., 2016). Unlike celebrity endorsers, local government officials are often unfamiliar to the public, so official familiarity is not a focus of this study. Likeability is an individual’s affinity for sources based on their physical appearance, personal traits (Kiecker and Cowles, 2002), or behavior (Erdogan, 1999; Munnukka et al., 2016), and is closely associated with friendliness (Bartneck et al., 2009; Nagel et al., 2021). Consequently, likeability can be subdivided into physical attractiveness and personality likeability, the latter being synonymous with friendliness. As officials are often perceived as high-power figures in social hierarchies (Zhu and Chang, 2023), demonstrating friendliness and similarity to the public can help cultivate a positive image. Based on these considerations, this study proposes three dimensions of official endorser attractiveness: physical attractiveness, interaction friendliness, and similarity with consumers.

Extensive literature has examined the factors of endorser credibility and attractiveness, focusing on their impact on consumer attitudes (Ha and Lam, 2016; Munnukka et al., 2016; Teng et al., 2020), trust (Qiu et al., 2021), risk perception (Deshbhag and Mohan, 2020), purchase intention (Ha and Lam, 2016; Kim and Kim, 2021; Masuda et al., 2022), and brand-related variables (Dwivedi and Johnson, 2013; Lou and Yuan, 2019) (refer to Table 2). However, few studies explored the sources of officials’ credibility and attractiveness, and separately explored the distinct roles of endorser credibility and attractiveness attributes on consumers.

**Table 2:** Studies about endorser’s attributes on endorsement effectiveness.

Independent variables	Dependent variables	Mediating variables	Endorse type	Source
Trustworthiness; Expertise; Attractiveness; Similarity; Liking; Familiarity; Match-up congruence with the brand/product	Purchase intention	Customer’s attitude toward brand	Celebrity endorsement	Ha and Lam (2016)
Attractiveness; Trustworthiness; Cause fit	Intention to purchase green cosmetics	Attitude toward green cosmetics	Celebrity endorsement	Lili et al. (2022)
Trustworthiness; Expertise; Attractiveness	Buyer intentions	Perceived risk	Celebrity endorsement	Deshbhag and Mohan (2020)
Attitude toward celebrity; Celebrity-brand fit; Celebrity expertise; Celebrity motive	Attitude toward Brand	Attitude toward endorsement	Celebrity endorsements	Bergkvist et al. (2016)
Expertise; Trustworthiness; Attractiveness; Likeability; Admirability	Intention to donate	Attitude toward charities	Celebrity endorsements	Wymer and Drollinger (2015)
Informative value; Entertainment value; Expertise; Trustworthiness; Attractiveness; Similarity	Brand awareness; Purchase intentions	Trust in branded posts	Influencer	Lou and Yuan (2019)
Source credibility: expertise, authenticity/trustworthiness; Source attractiveness: physical attractiveness, homophily/similarity	Loyalty to influencer; Product attitude; Purchase intention	Trust	Influencer	Kim and Kim (2021)
Perceived trustworthiness; Expertise; Uniqueness; Originality	OPRs (relationships with the corporation)	Mimicry desire; Altruistic CSR motives; CSR engagement	Social media influencers in CSR endorsement	Cheng et al. (2021)
Web celebrity popularity; Expertise; Interactivity; Appeal	Consumer impulse consumption	Pleasurable emotions; Evoking emotions	Web celebrity	Zhichao and Qien (2020)
Credibility: attractiveness, expertise, trustworthiness; Perceived congruence	Purchase intention	Brand attitude	Celebrity business ventures	Teng et al. (2020)
Expertise; Trustworthiness; Attractiveness	Consumer engagement: cognitive processing, affection, activation	Consumer trust: ability, benevolence, integrity	CEO	Qiu et al. (2021)
Trustworthiness; Expertness; Similarity; Attractiveness	Brand attitude	Product involvement; Attitude to advert	Peer endorser	Munnukka et al. (2016)

Given the significance of officials as endorsers in official live streaming, this study aims to apply two pivotal theories—source credibility theory and source attractiveness theory—to delve into the impact of officials’ credibility attributes and attractiveness attributes on consumers’ purchase intention and local brand awareness. In terms of intermediary mechanisms, official endorsements possess a unique characteristic: the backing of government credibility. Insufficient government credibility may erode public trust in officials’ promises and assurances during live streaming endorsements. The combined assurance of officials and government can notably bolster consumers’ perceived security. Therefore, this study explores the mediating role of consumers’ perceived security in the influence of officials’ credibility attributes on purchase intention and local brand awareness. Moreover, officials depart from their formal image to engage with consumers on online platforms, leveraging their personal charm to foster a closer rapport. This interaction often prompts positive evaluations and perceptions of enjoyment among consumers. Consequently, the study investigates the mediating role of consumers’ perceived enjoyment in the impact of officials’ attractiveness attributes on purchase intention and local brand awareness. Additionally, considering that officials predominantly endorse local brands, consumers’ regional backgrounds may influence their purchase intention and brand awareness. Preferences may diverge between local residents and out-shoppers, prompting the inclusion of consumer region as a moderating variable. Furthermore, traditional perceptions often associate officials with high-status individuals. Thus, consumers’ power distance belief may affect their attitudes toward official endorsements, for instance, consumers with lower power distance belief may seek direct interaction with officials online, aiming to bridge the perceived power gap and establish a more equitable relationship. Hence, this study incorporates consumers’ power distance belief as a moderating variable. In summary, this study aims to investigate the impacts of officials’ credibility attributes and attractiveness attributes on consumers’ purchase intention and local brand awareness. It also explores the mediating roles of perceived security and perceived enjoyment, as well as the moderating roles of consumer region and power distance belief.

## 3 Hypothesis development

### 3.1 The effect of official credibility attributes on perceived security

Online shopping involves inherent risks and security concerns (Tzavlopoulos et al., 2019). Consumers actively seek information to mitigate these risks and enhance their perception of security, considering factors such as security technologies, policies, company reputation, and endorsements from credible sources (Qalati et al., 2021). Unlike other forms, official endorsement provides consumers with a heightened sense of security. Therefore, this study focuses on perceived security to examine its impact on consumer responses. Building upon prior research, we define perceived security as the degree to which consumers regard products bought from officials as secure and reliable (Salisbury et al., 2001; Patel and Patel, 2018). It explores how an official’s credibility attributes namely expertise, trustworthiness, and government credibility, influence consumers’ perceived security in the context of official endorsements.

#### 3.1.1 The effect of official expertise on perceived security

Official expertise in local products is defined as the extent of their knowledge, experience, and skills related to these products (Hovland et al., 1953). Consumers rely on an endorser’s expertise in local products to assess their qualifications for making credible assertions in a specific domain (Kim and Kim, 2021). A local official with extensive knowledge of local products, including their unique characteristics, production techniques, usage, and cultural history, can assist consumers in choosing suitable and reliable products. In live streaming, officials’ expertise can diminish consumer risk perception and bolster their sense of security regarding products (Tong, 2018). Therefore, this study proposes the following hypothesis:

*H1*: Official expertise positively influences perceived security.

#### 3.1.2 The effect of official trustworthiness on perceived security

Trustworthiness encompasses honesty, sincerity, and believability (Ohanian, 1990). When promoting products, a trustworthy endorser provides objective information and guidance on product selection and usage. Generally, as independent intermediaries between merchants and consumers, officials’ endorsements of local products are not driven by personal economic interests, ensuring a degree of objectivity. Simultaneously, officials endorsing unqualified brands risk damaging their reputation and jeopardizing future promotional opportunities. This potential consequence heightens their caution and instills a more stringent approach when considering product endorsement. According to trust transfer theory (McKnight et al., 2002; Stewart, 2003), trust in officials can extend to the products they endorse (Chen et al., 2020), fostering psychological security (Yu and Yu, 2020) and enhancing consumer confidence. Therefore, this study proposes the following hypothesis:

*H2*: Official trustworthiness positively influences perceived security.

#### 3.1.3 The effect of government credibility on perceived security

Average consumers typically have limited direct interaction with local officials, leading to insufficient knowledge to assess their personalities and capabilities. In such scenarios, the credibility of the government plays a crucial role. Government credibility is defined as the extent of public trust in the government, encompassing citizens’ assessments of its capabilities and intentions. Research indicates that public trust in the government can reduce risk perceptions associated with using government services (Belanche et al., 2014), enhance the willingness to support government-organized events (Zhang and Dai, 2023), and stimulate the purchase intention of regional brand agricultural products (Xu et al., 2023). The distinctiveness of official endorsement stems from governmental influence; the implied government guarantee significantly boosts consumer confidence in local products. Therefore, this study proposes the following hypothesis:

*H3*: Government credibility positively influences perceived security.

### 3.2 The effect of official attractiveness attributes on perceived enjoyment

Perceived enjoyment, characterized by joy, delight, and pleasure (Akdim et al., 2022), is a primary motivator for media usage and technology adoption (Ma, 2021). Previous studies have demonstrated the positive impact of perceived enjoyment on consumer behavioral intentions (Wang et al., 2013; Wu and Santana, 2022). For instance, perceived enjoyment has been shown to positively correlate with engaging in social commerce on shopping websites (Zheng et al., 2020). This study proposes that an official’s attractiveness attributes, such as physical attractiveness, interaction friendliness, and similarity with consumers, can positively influence consumers’ perceived enjoyment of official endorsement.

#### 3.2.1 The effect of official physical attractiveness on perceived enjoyment

The adage “What is beautiful is good” is widely accepted (Chaker et al., 2019). Interacting with an attractive person tends to elicit happiness and positive emotions in individuals (Hazlett and Hoehn-Saric, 2000; Choi et al., 2020). Guo et al. (2022) suggested that a live streamer’s attractiveness positively correlates with consumers’ hedonic value in live streaming contexts. Similarly, highly attractive government officials are likely to receive more news coverage (Tsfati et al., 2010) and excellent promotion opportunities (Ling et al., 2019). An official endorser with a highly attractive physical appearance can captivate consumer interest and evoke feelings of pleasure, enjoyment, and other positive emotions when endorsing local products. Therefore, this study proposes the following hypothesis:

*H4*: Official physical attractiveness positively influences perceived enjoyment.

#### 3.2.2 The effect of official interaction friendliness on perceived enjoyment

Interaction friendliness, characterized by a pleasant and cheerful demeanor toward others, has positively influenced consumers’ positive emotions and experiences (Lee and Dubinsky, 2003). Demonstrating friendliness through smiling, gentleness, warmth, humor, and approachability can elicit positive consumer attitudes and evaluations (Liu et al., 2016). In everyday affairs, officials are often perceived as serious, authoritative, and distant in the public eye. To garner public support, some officials have begun actively manage their public image to be friendly, honest and intelligent through Internet platforms (Lalancette and Raynauld, 2019). In live streaming interactions, officials’ showing friendliness can reduce the psychological distance between officials and the public, enhancing consumers’ perceived enjoyment. Therefore, this study proposes the following hypothesis:

*H5*: Official interaction friendliness positively influences perceived enjoyment.

#### 3.2.3 The effect of official similarity on perceived enjoyment

McGuire (1985) defines similarity as the perceived resemblance between the endorser and consumer, encompassing demographic and ideological aspects such as values, interests, and preferences. People tend to favor others who are similar to them (Kandel, 1978; Hill and Stull, 1981), as this similarity not only fosters positive emotions toward others (Lee and Dubinsky, 2003) but also makes interactions feel more comfortable and enjoyable (Fu et al., 2018; Zhang et al., 2021). Additionally, government officials are often perceived as high-power figures within social hierarchies (Zhu and Chang, 2023). Unlike celebrity endorsers, consumers generally have limited understanding of and access to government officials. Suppose officials demonstrate their similarity to ordinary consumers through various means, such as using familiar “Internet” language, engaging in discussions on current popular topics, sharing common hobbies like singing or dancing, and even adopting similar attire with consumers. In that case, consumers are likely to be pleasantly surprised and form positive evaluations of the official endorsements. Therefore, this study proposes the following hypothesis:

*H6*: Official similarity with consumers positively influences perceived enjoyment.

### 3.3 The effect of perceived security and enjoyment on purchase intention

Consumers cannot accurately assess product quality without direct access to products online. These security concerns may deter and limit consumer engagement with e-commerce and online purchasing (Nepomuceno et al., 2014; Jiao et al., 2021). In official endorsement, when consumers feel secure, their intention to purchase is likely to be enhanced (Hartono et al., 2014). Therefore, this study proposes the following hypothesis:

*H7a*: Perceived security positively influences purchase intention.

Prior research has established that consumers’ positive emotions can significantly influence their purchase and other related behavioral intentions (Kim and Lennon, 2013; Lv et al., 2024). Fu et al. (2018) demonstrated that perceived enjoyment positively correlates with intention to purchase movie tickets online. Zheng et al. (2020) discovered that perceived enjoyment in social commerce enhances consumers’ purchase intentions. In the context of this study, when consumers experience enjoyment from official endorsements, this positive emotion can motivate them to purchase local products. Therefore, this study proposes the following hypothesis:

*H7b*: Perceived enjoyment positively influences purchase intention.

### 3.4 The effect of perceived security and enjoyment on local brand awareness

Memory theory posits that brand awareness correlates with the strength of the brand’s representation in memory (Keller, 1993; Loureiro, 2013). Brand awareness signifies consumers’ ability to recall, identify, or recognize a brand (Huang and Sarigöllü, 2012; Tan et al., 2021), serving as the initial step in forming brand associations in memory (Macdonald and Sharp, 2003), often indicated by “brand recognition memory” (Ye and Van Raaij, 2004). Developing local brands is crucial for the growth of the local economy (Pasquinelli et al., 2023), and official endorsements can play a pivotal and innovative role in this process.

As a positive factor, the perception of security can enhance consumers’ awareness of brands, thereby positively influencing their brand awareness (Chakraborty and Bhat, 2018). Prior research has shown a positive correlation between consumer trust and brand awareness. For instance, Al-Hawari (2011) demonstrated that the security of online services enhances brand awareness in retail banking. Lou and Yuan (2019) observed that consumer trust in influencers’ branded content positively impacts brand awareness. Therefore, this study proposes the following hypothesis:

*H8a*: Perceived security positively influences local brand awareness.

Research has established a link between positive emotions and memory. Yegiyan and Yonelinas (2011) discovered that positively arousing events can broaden memory effects. Madan et al. (2019) demonstrated that positive emotions distinctly affect memory, enhancing associative memory. In research by Wang and Li (2012), they found that perceived enjoyment of mobile services positively impacts brand awareness, subsequently driving purchase intention. Bae et al. (2020) revealed that perceived enjoyment in mixed reality experiences positively influences brand awareness in cultural heritage attractions. Lou and Yuan (2019) observed that the entertainment value of influencer-generated content positively correlates with brand awareness. Based on these studies, this study hypothesizes that when consumers find official endorsements enjoyable, it enhances their memory capacity and recall of local products, thereby increasing the likelihood of recognizing the local brand in various contexts. It is hypothesized that:

*H8b*: Perceived enjoyment positively influences local brand awareness.

### 3.5 The moderating role of consumer region

Based on geographical regions, consumers can be categorized into local consumers and out-shoppers. Local consumers are individuals residing in the same region as the officials. Notable differences exist between local consumers and out-shoppers. Local consumers’ preferences for local products are influenced by environmental concerns (Kareklas et al., 2014), product authenticity and quality (Carfora and Catellani, 2023), convenience, price (Birch et al., 2018), and a desire to support local communities (Memery et al., 2015). Out-shoppers primarily purchase local products due to their diversity, affordability, and superior quality (Dmitrovic and Vida, 2007). Consequently, consumers from different regions may exhibit distinct attitudes toward government officials and respond differently to official endorsements of local products. It is hypothesized that:

*H9*: Consumer region moderates H1–8, and the path patterns between local consumers and out-shoppers are different.

### 3.6 The moderating role of power distance belief

Power distance belief (PDB) is the degree to which individuals accept and expect power and wealth inequalities in society (Hofstede, 1984). Individuals with a high-power distance belief accept societal power inequalities and favor a stable social structure, believing that status should remain unchanged. Conversely, those with a low power distance belief prefer a more fluid social structure (Gao et al., 2016). Officials are often perceived as high-power figures within social hierarchies (Zhu and Chang, 2023), particularly in collectivist cultures such as China. This study proposes that individuals high in PDB are likely to avoid interactions with high-power officials, maintaining a distance that influences how official attributes affect consumer psychological and behavioral responses to official endorsements. It is hypothesized that:

*H10*: Power distance belief moderates H1–8, and the path patterns between consumers higher in PDB and consumers lower in PDB are different.

**Figure 1:**
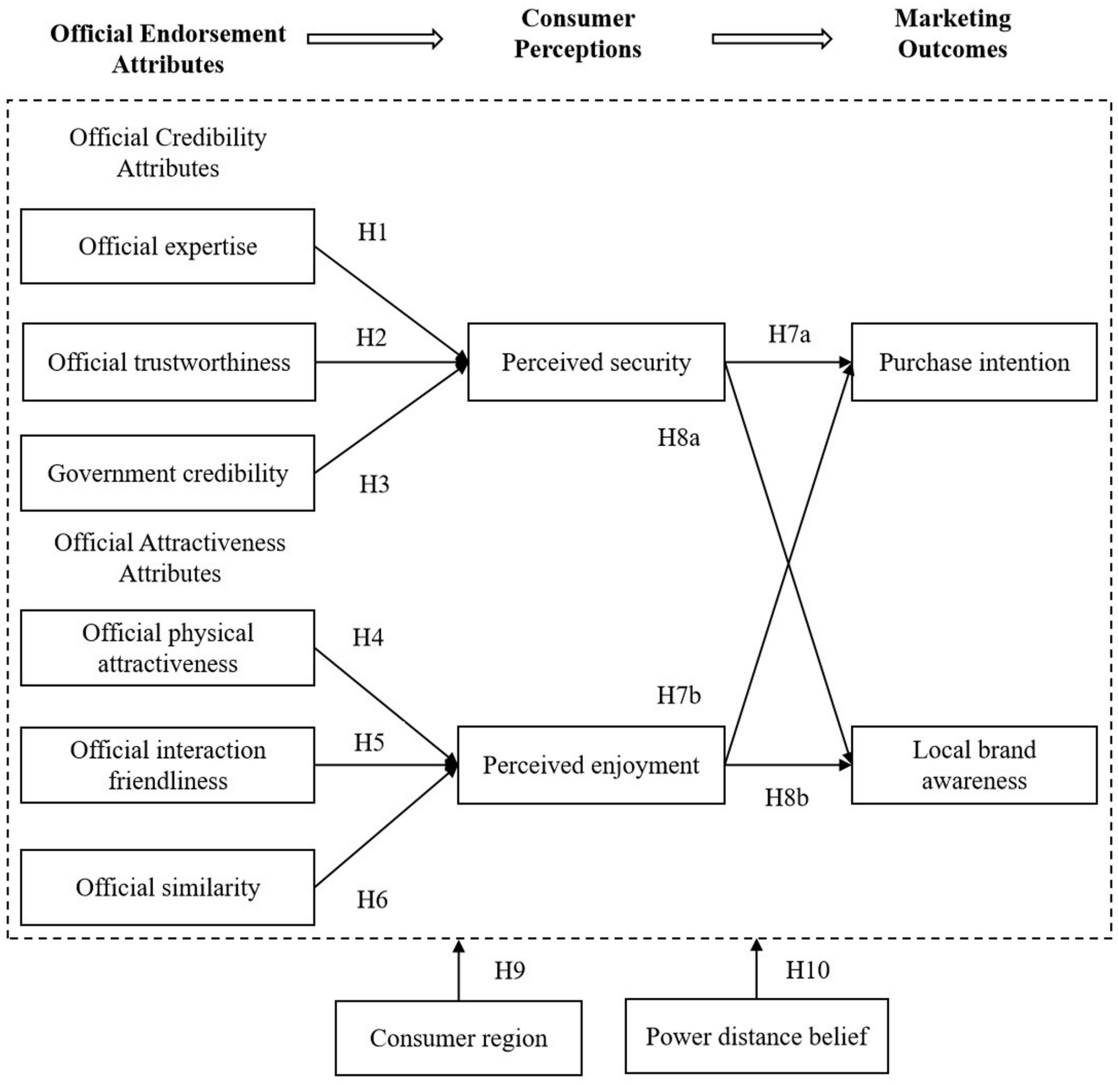
The hypotheses model.

The overview of these hypotheses is shown in the research model in Figure 1.

## 4 Method and analysis

### 4.1 Measurement

This study’s construct measurements were adapted and slightly modified from established scales to suit this study’s needs. Expertise was assessed using four items from Weismueller et al. (2020), trustworthiness through five items from Ohanian (1990), and government credibility via five items from Kim and Song (2020) and Napoli et al. (2014). Physical attractiveness was gaged using three items from Xu et al. (2020), interaction friendliness with four items from Crouch and Yetton (1988), and similarity with three items from Johnson and Grayson (2005). Additionally, perceived security was evaluated using three items from Aggarwal and Rahul (2018) and Salisbury et al. (2001), and perceived enjoyment with three items from Voss et al. (2003). Purchase intention was assessed with three items from Pavlou (2003), local brand awareness with four items from Dabbous and Barakat (2020), and power distance belief (PDB) with five items from Winterich and Zhang (2014). After collecting 112 questionnaires in the pretest, an ambiguous question item was removed from PDB. All items were evaluated using a seven-point Likert scale, ranging from 1 (“Strongly Disagree”) to 7 (“Strongly Agree”). The final scales were referred to Table 3.

**Table 3:** Measurement of constructs.

Construct	Item
Official expertise (Weismueller et al., 2020)	OE1: The official possesses a comprehensive knowledge of regional products.
	OE2: The official has much experience with regional products.
	OE3: The official is proficient in regional products.
	OE4: The official is an authority on regional products.
Official trustworthiness (Ohanian, 1990)	OT1: The official is dependable.
	OT2: The official is honest.
	OT3: The official is reliable.
	OT4: The official is sincere.
	OT5: The official is trustworthy.
Government credibility (Napoli et al., 2014; Kim and Song, 2020)	GC1: The government is trustworthy.
	GC2: The government is reliable.
	GC3: The government is convincing.
	GC4: The government is competent.
	GC5: The government is honest.
Official physical attractiveness (Xu et al., 2020)	OPA1: The official is handsome or beautiful.
	OPA2: The official is good-looking.
	OPA3: The official’s physical appearance is appealing.
Official interaction friendliness(Crouch and Yetton, 1988)	OF1: The official is friendly.
	OF2: The official is kind.
	OF3: The official is approachable and not arrogant during contact with customers.
	OF4: The official could foster a positive atmosphere in the live streaming space.
Official similarity (Johnson and Grayson, 2005)	OS1: The official and I have similar interests.
	OS2: The official and I have similar values.
	OS3: The official and I are similar in many ways.
Perceived security (Salisbury et al., 2001; Aggarwal and Rahul, 2018)	Purchasing products recommended by the officials,
	PS1: I feel safe.
	PS2: I feel secure.
	PS3: I feel at ease.
Perceived enjoyment (Voss et al., 2003)	For me, the official live streaming endorsement is
	PE1: Funny.
	PE2: Exciting.
	PE3: Enjoyable.
Purchase intention (Pavlou, 2003)	PI1: I’ll probably purchase products recommended by the official.
	PI2: I plan to purchase the product that the official has recommended.
	PI3: In the future, I plan to purchase products the official recommends.
Local brand awareness (Dabbous and Barakat, 2020)	LBA1: I can quickly recognize brands recommended by the official among other brands.
	LBA2: I am more familiar with brands recommended by the official than other brands.
	LBA3: Characteristics of brands recommended by the official come to my mind quickly.
	LBA4: It is easy to remember the names of brands I have seen on the official live streaming.
Power distance belief (Winterich and Zhang, 2014)	PDB1: 1 I think people in higher positions should make decisions without consulting those in lower positions.
	PDB2: I believe people in higher positions should keep their distance from those in lower positions.
	PDB3: I believe people in higher positions should not assign critical tasks to those in lesser positions.
	PDB4: I believe people in higher positions should not ask the opinions of those in lower positions too frequently.

### 4.2 Data collection and sample characteristics

Data collection occurred in China, necessitating the translation of the questionnaire into Chinese and subsequent back-translation into English for equivalence. The survey was conducted online through Credamo Credomo.com, a Chinese online survey platform. Ultimately, 594 valid responses were obtained. The demographic features of these respondents are provided in Table 4.

**Table 4:** Demographics of participants.

Characteristics		Frequency	Percentage (%)
Gender	Female	327	55.1
	Male	267	44.9
Age	<=24	96	16.2
	25–29	232	39.1
	30–39	222	37.4
	40–49	33	5.6
	≥ 50	11	1.9
Education	≤high school	21	3.5
	College	93	15.7
	Bachelor	413	69.5
	master	63	10.6
	doctorate	4	0.7
Monthly income (RMB)	<3,000	54	9.1
	3,000–5,000	97	16.3
	5,001–7,000	124	20.9
	7,001–9,000	162	27.3
	>9,000	157	26.4

## 5 Data analysis and results

This study introduced the structural equation modeling (SEM) as a technique to access the direct and indirect relationships between constructs. Unlike covariance-based SEM (CB-SEM), which focuses on maximizing the explained variance in the observed data, PLS-SEM prioritizes the prediction of latent variables (Hair et al., 2019). PLS-SEM is especially useful when dealing with small sample sizes, non-normal data, or complex models. It is particularly popular in social sciences, marketing, and other fields where complex relationships between variables need to be understood (Sun et al., 2019; Wang et al., 2021). In this study, PLS-SEM was chosen due to its suitability for exploratory research and its ability to predict model with data of non-normal distribution. Additionally, given the intention to compare participants’ regional background and power distance belief, PLS-SEM is more adaptable for smaller sample size compared to covariance-based SEM (Hair et al., 2019). Specifically, SmartPLS 3.0 was employed for testing the measurement and structural model in a dual-stage analysis. Finally, this study also applied SPSS 21.0 to address common method bias.

### 5.1 Common method bias

Initially, we incorporated OE, OT, GC, OPA, OIF, OS, PS, PE, PI, LBA, and PDB into the model to perform Harmon’s one-factor test, revealing that the first factor explained 35.10% of the covariance (Podsakoff et al., 2003). Subsequently, following Liang et al. (2007), we added a common method factor (CMF), assessed by all construct items in the model. The results indicated an average substantively explained variance of 0.679 for the items, an average method-based variance of 0.004, and a substantive to CMF variance ratio of approximately 169.75: 1. Furthermore, most loadings of the method factor were insignificant, indicating no apparent common method bias in this study.

### 5.2 Measurement model

Initially, we calculated composite reliability (CR), average variance extracted (AVE), Cronbach’s α (CA), and item loadings to evaluate the constructs’ internal reliability and convergent validity (refer to Table 5). The results demonstrated that all constructs’ item loadings exceeded 0.7, with CA, CR, and AVE values above 0.7 and 0.5, respectively, indicating strong reliability and convergent validity.

**Table 5:** Reliability and validity results.

Construct	Items	Standardized loadings	Cronbach’s alpha	CR	AVE
Official expertise	OE1	0.817	0.860	0.905	0.705
	OE2	0.851			
	OE3	0.875			
	OE4	0.815			
Official trustworthiness	OT1	0.759	0.834	0.883	0.601
	OT2	0.785			
	OT3	0.769			
	OT4	0.752			
	OT5	0.810			
Government credibility	GC1	0.781	0.823	0.876	0.586
	GC2	0.762			
	GC3	0.776			
	GC4	0.702			
	GC5	0.802			
Official physical attractiveness	OPA1	0.920	0.911	0.944	0.848
	OPA2	0.920			
	OPA3	0.923			
Official interaction friendliness	OIF1	0.806	0.770	0.853	0.592
	OIF2	0.813			
	OIF3	0.724			
	OIF4	0.730			
Official similarity	OS1	0.873	0.838	0.903	0.755
	OS2	0.847			
	OS3	0.887			
Perceived security	PS1	0.852	0.790	0.877	0.704
	PS2	0.844			
	PS3	0.821			
Perceived enjoyment	PE1	0.808	0.772	0.868	0.687
	PE2	0.844			
	PE3	0.834			
Purchase intention	PI1	0.836	0.811	0.888	0.725
	PI2	0.876			
	PI3	0.842			
Local brand awareness	LBA1	0.851	0.866	0.908	0.713
	LBA2	0.827			
	LBA3	0.849			
	LBA4	0.849			
Power distance belief	PDB1	0.817	0.819	0.880	0.648
	PDB2	0.815			
	PDB3	0.829			
	PDB4	0.756			

We then evaluated discriminant validity using three distinct methods. For the first check, all constructs’ item loadings aligned significantly with their intended constructs and not with others. The second check ensured that each construct’s square root of AVE exceeded its correlation with other constructs (refer to Table 6). Finally, all HTMT values were below the recommended threshold of 0.90 (refer to Table 7), indicating robust discriminant validity.

**Table 6:** Correlation coefficient matrix and square roots of AVEs (Fornell-Laker).

	GC	LBA	OE	OIF	OPA	OS	OT	PDB	PE	PI	PS
GC	0.769										
LBA	0.375	0.844									
OE	0.480	0.512	0.844								
OIF	0.530	0.451	0.544	0.772							
OPA	0.251	0.408	0.320	0.375	0.923						
OS	0.359	0.618	0.510	0.429	0.434	0.875					
OT	0.630	0.512	0.581	0.675	0.334	0.494	0.782				
PDB	−0.337	−0.228	−0.270	−0.321	−0.106	−0.181	−0.315	0.801			
PE	0.459	0.631	0.569	0.541	0.488	0.670	0.582	−0.232	0.833		
PI	0.464	0.590	0.552	0.608	0.373	0.595	0.613	−0.300	0.646	0.856	
PS	0.558	0.530	0.518	0.650	0.287	0.494	0.688	−0.299	0.553	0.633	0.836

OE, Official expertise; OT, Official trustworthiness; GC, Government credibility; OPA, Official physical attractiveness; OIF, Official interaction friendliness; OS, Official similarity with consumer; PS, Perceived security; PE, Perceived enjoyment; PI, Purchase intention; LBA, Local brand awareness.

**Table 7:** Heterotrait-Monotrait ratio of correlations (HTMT).

	GC	LBA	OE	OIF	OPA	OS	OT	PDB	PE	PI	PS
GC											
LBA	0.440										
OE	0.566	0.594									
OIF	0.665	0.548	0.660								
OPA	0.287	0.459	0.360	0.441							
OS	0.426	0.721	0.597	0.525	0.493						
OT	0.752	0.602	0.681	0.837	0.381	0.587					
PDB	0.409	0.269	0.319	0.402	0.122	0.218	0.380				
PE	0.568	0.765	0.692	0.687	0.577	0.825	0.717	0.288			
PI	0.562	0.700	0.656	0.761	0.433	0.714	0.739	0.365	0.804		
PS	0.688	0.642	0.627	0.833	0.339	0.607	0.842	0.371	0.702	0.788	

OE, Official expertise; OT, Official trustworthiness; GC, Government credibility; OPA, Official physical attractiveness; OIF, Official interaction friendliness; OS, Official similarity with consumer; PS, Perceived security; PE, Perceived enjoyment; PI, Purchase intention; LBA, Local brand awareness.

We conducted an additional analysis to assess multicollinearity. The collinearity assessment revealed that all indicators’ VIF values were below 3.33, indicating an absence of significant collinearity issues in this study.

### 5.3 Structural model

#### 5.3.1 Assessment of structural model

This study assessed the structural model using SmartPLS software. Compared to CB-SEM, which has standard fit indices, PLS-SEM emphasizes the predictive performance of the model rather than the degree of fit between the model and the data. Researchers often use metrics such as path coefficients, *R*^2^ (coefficient of determination), and *Q*^2^ (cross-validated redundancy) to assess the model’s predictive ability (Henseler and Sarstedt, 2013). In this study, the *R*^2^ value for perceived security, perceived enjoyment, purchase intention, and local brand awareness were 0.502, 0.537, 0.528, and 0.465, which means that the model explained 50.2% of the variance in perceived security, 53.7% in perceived enjoyment, 52.8% in purchase intention, and 46.5% in local brand awareness. In addition, the result of Blindfolding test showed that *Q*^2^ value for perceived security, perceived enjoyment, purchase intention, and local brand awareness were 0.344, 0.363, 0.373, and 0.325 respectively, which demonstrates a favorable predictive relevance (Sarstedt et al., 2014).

However, some researchers also suggest using values such as standardized root means square (SRMR), normed fit index (NFI), and root mean square error correlation (RMS_Theta) as measures of model fit to avoid misspecification in the model. Results of this study indicated that SRMR value was 0.067, which is below the threshold of 0.08, suggesting a good fit for the model. Although the NFI value was 0.807, slightly below the recommended threshold of 0.90, the difference was marginal. Additionally, the RMS_Theta value was 0.111, below the threshold of 0.12, further supporting the adequacy of the model fit (Henseler et al., 2016). Overall, the basic requirements for model fit had been satisfactorily met, indicating a good overall fit for the model.

#### 5.3.2 Structural equation modeling

The PLS-SEM was employed to estimate the path coefficients of the model. In terms of official credibility attributes, results showed that official expertise (β = 0.143, *p* < 0.05), trustworthiness (β = 0.480, *p* < 0.001), and government credibility (β = 0.184, *p* < 0.001) positively impacted consumers’ perceived security. In terms of official attractiveness attributes, official physical attractiveness (β = 0.180, *p* < 0.001), interaction friendliness (β = 0.246, *p* < 0.001), and similarity with consumers (β = 0.487, *p* < 0.001) positively influenced consumers’ perceived enjoyment. Additionally, perceived security (β = 0.398, *p* < 0.001) and enjoyment (β = 0.403, *p* < 0.001) significantly influenced consumers’ purchase intention. Both perceived security (β = 0.254, *p* < 0.001) and enjoyment (β = 0.472, *p* < 0.001) impacted local brand awareness.

**Figure 2:**
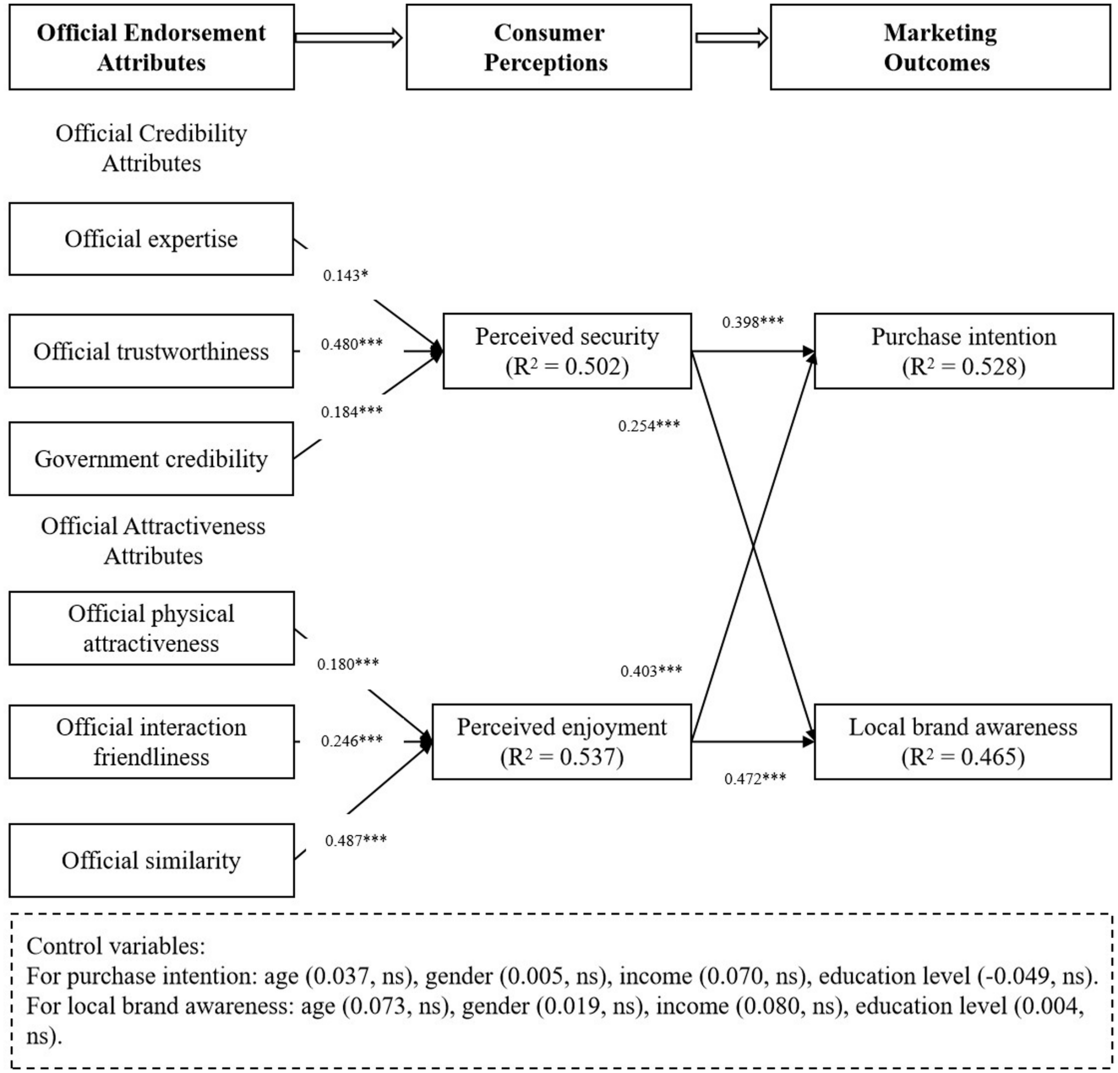
Results of the structural model.

Regarding control variables, the effects of age, gender, income and education level on purchase intention and local brand awareness were found to be insignificant. Thus, H1, H2, H3, H4, H5, H6, H7a, H7b, H8a, and H8b were all supported, as shown in Table 8 and Figure 2.

**Table 8:** Results of the path analyses.

Hypothesis	Path	Path coeffecients estimates	Standard Error	*t* statistics	*p*
H1	OE → PS	0.143^*^	0.065	2.193	0.027
H2	OT → PS	0.480^***^	0.067	7.203	0.000
H3	GC → PS	0.184^***^	0.053	3.483	0.000
H4	OPA → PE	0.180^***^	0.035	5.074	0.000
H5	OIF → PE	0.246^***^	0.033	7.475	0.000
H6	OS → PE	0.487^***^	0.035	13.862	0.000
H7a	PS → PI	0.398^***^	0.039	10.188	0.000
H7b	PS → LBA	0.254^***^	0.043	5.899	0.000
H8a	PE → PI	0.403^***^	0.038	10.561	0.000
H8b	PE → LBA	0.472^***^	0.041	11.533	0.000

^*^p < 0.05; ^**^p < 0.01; ^***^p < 0.001. OE, Official expertise; OT, Official trustworthiness; GC, Government credibility; OPA, Official physical attractiveness; OIF, Official interaction friendliness; OS, Official similarity with consumers; PS, Perceived security; PE, Perceived enjoyment; PI, Purchase intention; LBA, Local brand awareness.

### 5.4 Mediation analyses

This study further investigated the mediating roles of perceived security and enjoyment in the relationship between official attributes and both purchase intention and local brand awareness. Regarding the mediating role of perceived security in the impact of official credibility attributes on marketing outcomes, perceived security fully mediated the relationship between official trustworthiness and government credibility, as well as both purchase intention and local brand awareness. However, the mediating role of perceived security in the relationship between official expertise and purchase intention (*p* = 0.057) and local brand awareness (*p* = 0.088) was not statistically significant.

Concerning the mediation of perceived enjoyment in the influence of official attractiveness attributes on marketing outcomes, perceived enjoyment fully mediated the impact of official physical attractiveness on purchase intention and partially mediated its effect on local brand awareness. Perceived enjoyment partially mediated the relationship between official interaction friendliness and purchase intention while fully mediating its impact on local brand awareness. Moreover, perceived enjoyment partially mediated official similarity’s effect on purchase intention and local brand awareness (refer to Table 9).

**Table 9:** Results of mediating effects.

Paths	Direct effect LLCI: ULCI		Indirect effect LLCI: ULCI		Results
OE → PS → PI	0.082^*^	(0.009: 0.162)	0.030	(0.005: 0.064)	None
OE → PS → LBA	0.131^**^	(0.033: 0.231)	0.023	(0.003: 0.055)	None
OT → PS → PI	0.069	(−0.035: 0.168)	0.098^***^	(0.051: 0.153)	Full
OT → PS → LBA	0.053	(−0.041: 0.154)	0.076^**^	(0.033: 0.123)	Full
GC → PS → PI	0.002	(−0.097: 0.109)	0.038^**^	(0.016: 0.063)	Full
GC → PS → LBA	0.011	(−0.072: 0.091)	0.029^*^	(0.010: 0.058)	Full
OPA → PE → PI	−0.009	(0.075: 0.056)	0.039^***^	(0.020: 0.063)	Full
OPA → PE → LBA	0.084^*^	(0.003: 0.165)	0.043^**^	(0.021: 0.070)	Partial
OIF → PE → PI	0.185^***^	(0.102: 0.272)	0.054^***^	(0.029: 0.082)	Partial
OIF → PE → LBA	−0.047	(−0.146: 0.051)	0.059^***^	(0.031: 0.090)	Full
OS→ PE → PI	0.199^***^	(0.123: 0.273)	0.106^***^	(0.061: 0.158)	Partial
OS→ PE → LBA	0.276^***^	(0.179: 0.375)	0.117^***^	(0.066: 0.168)	Partial

^*^p < 0.05; ^**^p < 0.01; ^***^p < 0.001. Level of confidence = 95%. LLCI, Lower limit of the confidence interval; ULCI, Upper limit of the confidence interval; OE, Official expertise; OT, Official trustworthiness; GC, Government credibility; OPA, Official physical attractiveness; OIF, Official interaction friendliness; OS, Official similarity with consumers; PS, Perceived security; PE, Perceived enjoyment; PI, Purchase intention; LBA, Local brand awareness.

### 5.5 Moderation analyses based on consumer region

In this study, 134 participants, representing 22.6%, shared the same region as the official endorsers, while 460 participants, constituting 77.4%, were out-shoppers, reflecting a realistic distribution. To examine the impact of consumer region, we initially conducted an independent-samples t-test to investigate the differences between local consumers and out-shoppers. According to Table 10, local consumers exhibited higher perceptions of security, enjoyment, purchase intention, and local brand awareness than out-shoppers.

**Table 10:** Independent-samples’ *t*-test for means of “local” and “out-shopper” groups.

Construct/Group	Local	Out-shopper	Mean difference	*p*
PS	6.2711	6.1319	0.13926	0.033
PE	5.7313	5.5123	0.21902	0.006
PI	6.0050	5.7319	0.27309	0.000
LBA	5.7985	5.4418	0.35666	0.000

^*^p < 0.05; ^**^p < 0.01; ^***^p < 0.001; n.s., not significant. OE, Official expertise; OT, Official trustworthiness; GC, Government credibility; OPA, Official physical attractiveness; OIF, Official interaction friendliness; OS, Official similarity with consumers; PS, Perceived security; PE, Perceived enjoyment; PI, Purchase intention; LBA, Local brand awareness.

We then conducted a multi-group analysis to compare local consumers with out-shoppers (Matthews, 2017) (refer to Table 11). The results indicated that: (1) For local customers compared to out-shoppers, the correlation between government credibility and perceived security was noticeably larger (β_local consumer_ = 0.437, *p* < 0.001; β_out-shopper_ = 0.146, *p* < 0.05; β_difference_ = 0.291, *p* < 0.01). (2) Out-shoppers had a far stronger association between official interaction friendliness and perceived enjoyment than local consumers (β_local consumer_ = 0.071, *p* > 0.05; β_out-shopper_ = 0.307, *p* < 0.001; β_difference_ = −0.236, *p* < 0.01). (3) Local consumers had a considerably stronger correlation between perceived enjoyment and local brand awareness compared to out-shoppers (β_local consumer_ = 0.650, *p* < 0.001; β_out-shopper_ = 0.455, *p* < 0.001; β_difference_ = 0.195, *p* < 0.05). As a whole, hypothesis 9 was supported partially.

**Table 11:** Results of path coefficients between local consumers and out-shoppers.

Hypothesis		Path coefficients		
		Local consumer	Out-shopper	Difference
H1	OE - > PS	0.071	0.162^*^	−0.091
H2	OT - > PS	0.298^**^	0.500^***^	−0.202
H3	GC - > PS	0.437^***^	0.146^*^	0.291^**^
H4	OPA - > PE	0.223^**^	0.173^***^	0.050
H5	OIF - > PE	0.071	0.307^***^	−0.236^**^
H6	OS - > PE	0.579^***^	0.445^***^	0.134
H7a	PS - > PI	0.395^***^	0.394^***^	0.001
H7b	PE - > PI	0.363^***^	0.437^***^	−0.074
H8a	PS - > LBA	0.175^*^	0.284^***^	−0.109
H8b	PE - > LBA	0.650^***^	0.455^***^	0.195^*^

^*^p < 0.05; ^**^p < 0.01; ^***^p < 0.001; n.s., not significant. OE, Official expertise; OT, Official trustworthiness; GC, Government credibility; OPA, Official physical attractiveness; OIF, Official interaction friendliness; OS, Official similarity with consumers; PS, Perceived security; PE, Perceived enjoyment; PI = Purchase intention, LBA = Local brand awareness.

### 5.6 Moderation analyses based on power distance belief

Participants were categorized into three groups based on their power distance belief scores: low (*N* = 201), medium (*N* = 196), and high in PDB (*N* = 197). An independent-sample *t*-test was conducted to investigate the differences among consumers low, medium, and high in PDB. Table 12 reveals that consumers low in PDB own higher levels of perceived security, perceived enjoyment, purchase intention, and local brand awareness than consumers medium and high in PDB.

**Table 12:** Independent-sample *t*-test for PDB means of low and high groups.

Construct/Group	Low in PDB	Medium in PDB	High in PDB	Low vs. Medium	Medium vs. High	Low vs. High
PS	6.4262	6.1020	5.9560	0.32416^***^	0.14603^*^	0.47020^***^
PE	5.7977	5.5204	5.3621	0.27727^**^	0.15831^*^	0.43558^***^
PI	6.1244	5.6973	5.5516	0.42710^***^	0.14567	0.57277^***^
LBA	5.8097	5.5089	5.2424	0.30077^***^	0.26654^**^	0.56732^***^

^*^p < 0.05; ^**^p < 0.01; ^***^p < 0.001; n.s., not significant. OE, Official expertise; OT, Official trustworthiness; GC, Government credibility; OPA, Official physical attractiveness; OIF, Official interaction friendliness; OS, Official similarity with consumers; PS, Perceived security; PE, Perceived enjoyment; PI, Purchase intention; LBA, Local brand awareness.

Subsequently, a multi-group analysis was conducted to compare consumers low, medium, and high in PDB (refer to Table 13). The pairwise comparison found that: (1) When comparing consumers low in PDB with consumers medium in PDB, there was no significant difference in all path coefficients. (2) When comparing consumers medium in PDB and high in PDB, the correlation between official similarity and perceived enjoyment was considerably more pronounced among consumers with medium PDB scores than those with high PDB scores (β_medium in PDB_ = 0.528, *p* < 0.001; β_high in PDB_ = 0.353, *p* < 0.001; β_difference_ = 0.176, *p* < 0.05). (3) When comparing consumers low in PDB and high in PDB, the correlation between official similarity and perceived enjoyment was also considerably more pronounced among consumers with low PDB scores than consumers high in PDB (β_low in PDB_ = 0.570, *p* < 0.001; β_high in PDB_ = 0.353, *p* < 0.001; β_difference_ = 0.217, *p* < 0.05). Overall, for consumers with lower PDB, official similarity exerted a greater influence on perceived enjoyment. In conclusion, hypothesis 10 received partial support.

**Table 13:** Path coefficient comparisons for consumers with low, medium, and high PDB.

Hypothesis/Group		Low in PDB	Medium in PDB	High in PDB	Low vs. Medium	Medium vs. High	Low vs. High
H1	OE - > PS	0.140	0.175	0.077	−0.035	0.097	0.063
H2	OT - > PS	0.348^***^	0.538^*^	0.492^***^	−0.190	0.046	−0.144
H3	GC - > PS	0.235^**^	0.086	0.256^***^	0.149	−0.171	−0.022
H4	OPA - > PE	0.152^*^	0.147^**^	0.245^***^	0.005	−0.098	−0.093
H5	OIF - > PE	0.151^*^	0.280^***^	0.292^***^	−0.129	−0.012	−0.141
H6	OS - > PE	0.570^***^	0.528^***^	0.353^***^	0.042	0.176^*^	0.217^*^
H7a	PS - > PI	0.355^***^	0.398^***^	0.315^***^	−0.043	0.083	0.040
H7b	PE - > PI	0.426^***^	0.447^***^	0.429^***^	−0.021	0.018	−0.003
H8a	PS - > LBA	0.235^***^	0.338^***^	0.165^*^	−0.102	0.173	0.070
H8b	PE - > LBA	0.566^***^	0.430^***^	0.474^***^	0.136	−0.044	0.092

^*^p < 0.05; ^**^p < 0.01; ^***^p < 0.001; n.s., not significant. OE, Official expertise; OT, Official trustworthiness; GC, Government credibility; OPA, Official physical attractiveness; OIF, Official interaction friendliness; OS, Official similarity with consumers; PS, Perceived security; PE, Perceived enjoyment; PI, Purchase intention; LBA, Local brand awareness.

## 6 Discussion and conclusion

### 6.1 Discussion

Live streaming has emerged as the premier platform for entertainment, interaction and e-commerce. Given its widespread polularity, government officials are encouraged to leverage this platform to utilize their inherent authority and personal charisma to foster stronger connections with the public, and stimulate local economic growth. Consequently, this study concentrates on the influence of official endorsements in live streaming, examining how the credibility and attractiveness attributes of official endorsers influence consumers’ purchase intention and local brand awareness. It further investigates the mediating roles of perceived security and perceived enjoyment, along with the moderating effects of consumer region and power distance belief.

Firstly, addressing the inherent risks associated with live streaming shopping, this research integrates the concept of consumers’ perceived security. It explores how the credibility attributes of official endorsers—namely official expertise, trustworthiness, and government credibility—affect consumer perceived security. Findings indicate that endorsers with high levels of expertise and trustworthiness significantly enhance consumers’ perceived security, corroborating earlier studies which have shown that an endorser’s expertise and trustworthiness effectively boost consumer trust and mitigate risk perceptions (Chen et al., 2020; Deshbhag and Mohan, 2020; Qiu et al., 2021). Additionally, this study introduces government credibility as another source of official credibility and demonstrates its effect on consumers’ security perception, which aligns with previous research demonstrating that government credibility can diminish the public’s risk perception when engaging with e-government services (Bélanger and Carter, 2008). These insights significantly broaden the understanding of credibility sources within official endorsements (Kim and Kim, 2021).

Secondly, recognizing the entertainment value of live streaming, this study emphasizes perceived enjoyment, investigating how the attractiveness attributes of officials—physical attractiveness, interaction friendliness, and similarity with consumers—affect consumer perceived enjoyment. This investigation supports existing literature that associates endorser attractiveness with increased consumer arousal (Xu et al., 2020), pleasure (Zhichao and Qien, 2020), and enjoyment value (Guo et al., 2022). Notably, this study contributes to the discussion by breaking down the attractiveness traits of endorsers more clearly and examining their combined impact on perceived enjoyment (Teng et al., 2020; Masuda et al., 2022). Through this approach, the research adds to the sources of endorser attractiveness and highlights the positive emotional impacts these attributes create for consumers.

Third, this study explores the mediating role of perceived security in the relationship between official credibility attributes and both purchase intention and local brand awareness. While trust is often emphasized in endorsement research (Lou and Yuan, 2019; Kim and Kim, 2021), perceived security has not been as thoroughly investigated. This research identifies perceived security as a mediating mechanism, finding that official trustworthiness and government credibility enhance consumers’ perceived security, which in turn positively influences purchase intention and local brand awareness. This aspect of the study not only clarifies how officials’ credibility attributes trigger positive consumer responses but also expands the scope of research on perceived security. However, the study also notes that the mediating role of perceived security in the impact of official expertise on purchase intention and local brand awareness was insignificant. We speculate that other mediating variables, such as perceived utility value, might influence the impact of expertise on consumer responses, as suggested by Guo et al. (2022), who noted that expertise affects purchase intention through perceived utilitarian and hedonic values.

Fourth, the role of perceived enjoyment as a mediator in the relationship between official attractiveness attributes and both purchase intention and local brand awareness is discussed. Previous studies have demonstrated the positive effects of hedonic value on purchase intention (Park and Lin, 2020; Guo et al., 2022) and brand awareness (Dabbous and Barakat, 2020), yet the impact of endorser attractiveness attributes on consumers’ perceived enjoyment has been less explored. This study establishes a link between officials’ attractiveness attributes and consumer responses, using perceived enjoyment as a mediator. It highlights that officials’ physical attractiveness, interaction friendliness, and similarity to consumers boost consumers’ perceived enjoyment, which can subsequently enhance purchase intention and amplify local brand awareness.

Fifth, this research compares the differing responses between local consumers and out-shoppers. Birch et al. (2018) identified evaluative and preferential disparities between these groups. The findings of this study further reveal that: (1) Government credibility has a reduced impact on perceived security among out-shoppers, likely due to their limited understanding of local governments and their credibility levels; (2) Official interaction friendliness has a greater impact on perceived enjoyment for out-shoppers, indicating that these consumers highly value the hospitality of local officials, aligning with Freire (2009), who highlighted the significance of local friendliness in destination-brand building; (3) Perceived enjoyment influences local brand awareness more significantly among local consumers, suggesting that they hold positive attitudes and evaluations toward local officials’ endorsement activities. Although these differences are not universal, they indicate that local consumers and out-shoppers exhibit distinct attitudes and preferences toward official endorsements in certain respects.

Lastly, in conjunction with the power image of officials, this study examines how consumers with different power distance belief respond to official endorsements. Winterich et al. (2018) found that individuals with low power distance belief seek closeness and equality with high-status figures, whereas those with high power distance belief prefer to maintain a distance. The current study corroborates these findings, demonstrating that for consumers with lower power distance belief, the effect of official similarity on perceived enjoyment is more pronounced.

### 6.2 Theoretical contributions

This study makes several crucial contributions to the field as follows:

First, it provides a theoretical framework for understanding the impact of official endorsement by demonstrating how the credibility and attractiveness attributes of officials influence consumer purchase intention and local brand awareness through the mediators of perceived security and enjoyment. While existing research primarily focuses on celebrities, peers, experts, and entrepreneur endorsers (Munnukka et al., 2016; Teng et al., 2020; von and Breuer, 2020; Choi et al., 2023), this research uniquely centers on official endorsements, examining not only purchase intention but also the enhancement of local brand awareness as marketing outcomes. This approach represents a significant and valuable addition to the endorsement literature.

Secondly, this study enriches the endorsement literature by expanding the source credibility model and the source attractiveness model (Schimmelpfennig and Hunt, 2020; Halder et al., 2021). Regarding the source credibility model, previous studies have largely focused on source expertise and trustworthiness (Ohanian, 1990; O’keefe, 2015), exploring their effects on consumer trust (Lou and Yuan, 2019; Lu and Chen, 2021), risk perception (Hussain et al., 2017; Deshbhag and Mohan, 2020), attitude (Wang and Scheinbaum, 2018; Teng et al., 2020), and purchase intention (Lou and Yuan, 2019; Chen et al., 2020). This research enhances the source credibility model by incorporating government credibility as a key dimension and exploring its influence on consumers’ perceived security. Concerning the source attractiveness model, this study divides the sources of officials’ attractiveness in live streaming into physical attractiveness, interaction friendliness and similarity with consumers, providing a nuanced view of source of attractiveness in official endorsement context (Qiu et al., 2021; Lili et al., 2022). While prior research has mainly focused on the effects of endorser attractiveness on consumer attitudes or trust (Wang and Scheinbaum, 2018; Kim and Kim, 2021), this study advances the theoretical framework by emphasizing how official attractiveness attributes impact consumer perceived enjoyment.

Third, this study elucidates the different attitudes toward official endorsement between local consumers and out-shoppers, especially in how government credibility influences perceived security, how official interaction friendliness enhances perceived enjoyment, and how perceived enjoyment subsequently affects local brand awareness, which expands on the recognized differences in evaluations and preferences between local consumers and out-shoppers as outlined by Birch et al. (2018). Furthermore, building on findings by Winterich et al. (2018) regarding power distance beliefs, this study delves deeper by demonstrating that the similarities between consumers and officials can increase perceived enjoyment among consumers with low power distance beliefs. This adds a nuanced understanding of how consumer-official dynamics influence endorsement effectiveness based on cultural dimensions.

Lastly, this research introduces a new dimension to the existing body of knowledge concerning government and officials, which previously focused on aspects like local government policies (Chai et al., 2023) and attributes (Xu et al., 2023), as well as officials’ political viewpoints (Clayman, 2017) and behaviors such as information disclosure (Choi, 2018). By specifically focusing on the endorsement behavior of government officials toward local products and brands, this study enriches our understanding of their role in a marketing context, highlighting how official endorsements can influence consumer perceptions and behaviors.

### 6.3 Managerial implications

Official endorsement offers a unique strategy for marketing local brands. This study delivers crucial insights for marketers on the practical application of official endorsement in marketing strategies. Firstly, to boost consumer purchase intention and local brand awareness, marketers must recognize the importance of perceived security and enjoyment. Alongside highlighting the security of official endorsements, eliciting emotional enjoyment in official live streaming is also crucial. Secondly, the study underscores the role of official credibility attributes—expertise, trustworthiness, and government credibility—in enhancing consumers’ security perception. Marketers should emphasize officials’ knowledge of local products, their trustworthiness and sincerity, and the reliability guaranteed by government support. Thirdly, the study highlights that an official’s physical attractiveness, interaction friendliness, and similarity with consumers drive perceived enjoyment in endorsements. Marketers can improve an official’s appeal, foster a friendly image, and establish consumer rapport through shared language and similar attire. Finally, acknowledging diverse consumer scenarios, marketers should adapt the attributes of official endorsers accordingly; for example, accentuating an official’s friendly image may be especially effective in attracting out-shoppers.

## 7 Limitations and future research directions

This study is subject to some limitations. Firstly, our exploration of the influence of official endorser attributes on consumer behavior primarily draws from source credibility and attractiveness theory, neglecting a thorough examination of the characteristics inherent to endorsed products and brands (e.g., product quality, brand image), as well as the iamge fit between official endorsers and these entities. Future research endeavors could expand the scope of inquiry to encompass these facets, offering a more comprehensive understanding of the official endorsement. Secondly, our reliance on the online questionnaire survey method presents certain constraints. For instance, the data are sourced from a single channel and are self-reported, potentially compromising objectivity and validity. To address this, forthcoming studies could consider adopting a mixed-methods approach that integrates quantitative and qualitative research methodologies, such as interviews and text analysis. This diversified approach holds promise for enhancing the study’s validity and robustness. Thirdly, this study focused on collecting data from Chinese consumers, limiting the universality of the research conclusions to some extent. This study selected data from China is mainly due to the widespread use of official live streaming endorsement in China. However, it is undoubtedly that the global expansion of live streaming e-commerce demonstrating the potentional of official endorsements beyond China. Government officials may, for instance, promote domestic products and brands internationally, effectively representing their country’s offerings in foreign markets. Therefore, future studies could make further exploration in diverse national contexts. Additionally, this study concentrated on the purchase intention for local products, omitting the impact of official endorsements on local tourism resources and consumer visitation intentions. Future research could investigate these overlooked aspects.

## Data availability statement

The raw data supporting the conclusions of this article will be made available by the authors, without undue reservation.

## Ethics statement

Ethical review and approval were not required for the study on human participants in accordance with the local legislation and institutional requirements. The studies were conducted in accordance with the local legislation and institutional requirements. Written informed consent for participation was not required from the participants or the participants’ legal guardians/next of kin in accordance with the national legislation and institutional requirements.

## Author contributions

GC: Conceptualization, Funding acquisition, Project administration, Resources, Supervision, Validation, Writing – original draft, Writing – review & editing. WL: Conceptualization, Data curation, Formal analysis, Investigation, Methodology, Project administration, Software, Writing – original draft, Writing – review & editing. MH: Data curation, Investigation, Writing – original draft. LL: Conceptualization, Writing – original draft.
